# Extraction and Characterization of Collagen with Cost-Effective Method from Human Placenta for Biomedical Applications

**DOI:** 10.29252/wjps.8.3.352

**Published:** 2019-09

**Authors:** Ali Karami, Hamid Tebyanian, Reza Sayyad Soufdoost, Ebrahim Motavallian, Aref Barkhordari, Mohammad Reza Nourani

**Affiliations:** 1 Nanobiotechnology Research Center, Baqiyatallah University of Medical Sciences, Tehran, Iran;; 2 Research Center for Prevention of Oral and Dental Diseases, Baqiyatallah University of Medical Sciences, Tehran, Iran;; 3 Imam Khomeini Clinic of Dentistry, Tehran, Iran;; 4 Department of General Surgery, Faculty of Medicine, Baqiyatallah University of Medical Sciences, Tehran, Iran

**Keywords:** Collagen, Huma, Placenta, Extraction, Characterization

## Abstract

**BACKGROUND:**

Collagen is the main product in pharmaceutics and food industry with a high demand. Collagen can be extracted from several tissues such as skin, bone and tendon, etc. Collagen can be used in tissue engineering researches as a substrate of wound healing and nerve regeneration. Extraction methods of collagen are various with different purities. In this research, we aimed to extract collagen from human placenta with a modified method.

**METHODS:**

This modified approach was used for extracting of collagen from human placenta with acetic acid and NaCl treatment using different concentrations.

**RESULTS:**

SDS page showed three different bands that reflected two alpha-chains and one beta-chain with molecular weights of 102, 118 and 220 kDa, respectively. There was no significant difference between extracted collagen from human placenta and standard collagen in western blot analysis.

**CONCLUSION:**

It was concluded that human placenta can be an alternative source of collagen with high purity for biomedical applications such as tissue engineering, stem cell therapy and research.

## INTRODUCTION

Collagen can be found in all multicellular animals.^1^^,^^2^ Collagen is a significant factor in vertebrates and invertebrates structure and collagen is the main component protein of skin, tendons, cartilage, bones and tissues in general.^2^ Collagen molecules (280 nm long and 360,000 Da) have become stable by hydrogen bonds and intermolecular bonds.^3^ Also, they have three helical polypeptide chains (1000 amino acids) which are known as α chain. The triple helix molecules have terminal globular domains named procollagen which becomes stable by hydrophobic and electrostatic interactions.^4^^,^^5^

There is various type of collagen (29 different types) in vertebrates^5^ classified according to their structure into striatum (fibrous), non-fibrous (network forming), and microfibrillar (filamentous).^4^^,^^6^^-^^8^ Connective tissue has different types of collagen which have various forms in tissues of all species of multicellular organisms.^4^^,^^9^ Collagen can be extracted from numerous animal species and it is usually derived from animal skin, tendon, cartilage and bone as the main sources of collagen. Some researchers have investigated on different approaches of collagen extraction from different animal sources, such as fish and birds.^10^^-^^12^

Collagen as a biomaterial has high protein content, and functional properties (water absorption capacity, gel formation, stabilizing emulsions) and also, it has an extensive use in drug and food industries.^13^^,^^14^ Collagen can be used in biomedical, pharmaceutical and tissue engineering fields and also, it can be used in clinical settings as a substitute for human skin, blood vessels and ligaments.^6^^,^^7^^,^^15^^,^^16^ Collagen can be extracted by chemical and enzymatic hydrolysis.^17^ Chemical hydrolysis is usually used more in industry, but biological procedures which use enzymes are more hopeful.^18^

Furthermore, enzymatic procedures make less waste and decrease the processing time, but they are not cost effective. So it is important to remove numerous covalent intra- and inter-molecular cross-links which create the procedure quite complex.^11^ Safe and cost-effective extracted collagen have been requested in this decade and collagen applications have been increased for clinical and non-clinical usage. In this study, the modified approach of human placenta collagen extraction was investigated with a cost-effective approach for clinical applications such as cell culture, tissue engineering, cell therapy and research, industrial use (pharmaceutical applications), etc. 

## MATERIALS AND METHODS

All chemicals were purchased from Sigma Chemical Co. (St. Louis, MO, USA) including NaOH, Butyl Alcohol, Acetic Acid, NaCl and Sodium Phosphate. The human placenta was obtained from a hospital with approval from Ethical Committee of Baqiyatallah University of Medical Sciences, Tehran, Iran. Residual blood was removed and cleaned samples were washed with distilled water. All the preparation procedures were performed at 4˚C with a continuous stirring. 

**Fig. 1:**
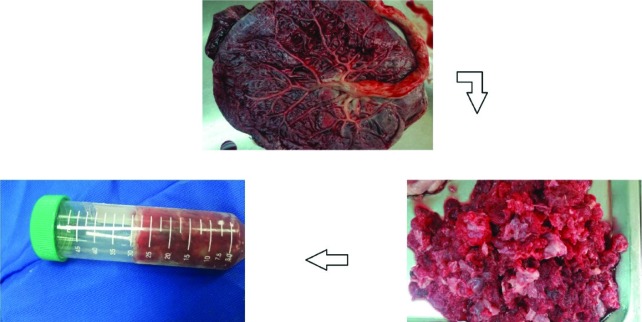
The process of human placenta for collagen extraction

The washed samples were chopped (1-2 cm). To remove non-collagenous proteins, the placenta was mixed with 0.1 N NaOH at a sample/alkali solution at ratio of 1:10 (w/v). The mixture was stirred for 6 h. The alkali solution was changed every 2 h. Then, they were placed in polyethylene bags and stored at -20˚C until used. All reagents used in this study were an analytical grade (Figure 1). Sampled were thawed and washed with cold distilled water in stirring condition at 4˚C for 2 h. A 10 % butyl alcohol was used for removing extra fat (1:8 (w/v)- overnight, fresh solvent every 12 h). 

Samples were rinsed with distilled water, then 0.06 M acetic acid (1:25(w/v) was added overnight. The solution was filtered with cheese cloth (four times). NaCl was added to the solution (final concentration was 3 M, pH=7) in order to precipitate collagen (cold room, overnight, stirring). Then, the precipitate was centrifuged at 15000 g for 30 min, the supernatant was discarded and the remaining was dissolved in 2% acetic acid. It was transferred to dialysis tubing cellulose membranes and stirred in distilled water (refreshed every 8 h) for 5 days and finally, freeze-dried. 

The solubility was detected by Montero, Jimenez-Colmenero, and Borderias approach.^19^ The obtained collagen was dissolved in 0.6 M acetic acid to get 3 mg/ml as final concentration (stirring, cold water 4ºC). Totally, 5 ml of collagen (5 mg/ml) in 0.6 M acetic acid were blended with 5 ml of NaCl in 0.6 M acetic acid at several concentrations (0%, 2%, 4%, 6%, 8%, 10% and 12% (w/v)) and stirred in cold room (4ºC) for 40 min and then, centrifuged at 15000 g at 4ºC for 45 min. The supernatant was evaluated. The bovine serum albumin was used as a standard.^19^

SDS–PAGE was performed by the method of Laemmli (1970).^20^ The collagen samples were dissolved in 0.02 M sodium phosphate containing 1% SDS and 3.5 M urea (pH=7.2) with continuous stirring at room temperature. The combinations were centrifuged at 8500 g for 5 min at room temperature to remove undissolved debris. Solubilized samples were mixed at 1:1 (v/v) ratio with the sample buffer (0.5 M Tris–HCl, pH=6.8, containing 4% SDS, and 20% glycerol) in the presence or absence of 10% beta-mercaptoethanol (BME). Samples were loaded onto the PAGEL, compact precast gel (5% gel) and subjected to electrophoresis at a constant current of 20 mA/gel using a compact-PAGE apparatus. Gel was stained after electrophoresis with 0.05% Coomassie Blue R-250 (w/v) in 15%methanol (v/v) and 5% acetic acid (v/ v) and then, it was destained with 30% methanol (v/v) and 10% acetic acid (v/v).^20^^-^^23^

Western blot analysis (antigen labeling) was performed according to Timmons and Dunbar (1990). ^24^ The collagen was transferred (10 V for 50 min) with the Trans-Blot Semi-Dry Transfer Cell (Bio-Rad Laboratories, Hercules, CA 94547) to an Immun Blot polyvinylidene fluoride membrane (Bio-Rad Laboratories, Hercules, CA, 94547, 0.2 μm). The primary antibody used was Anti-Collagen I antibody (C9791), whereas the secondary antibody was Goat Anti-Rabbit IgG (ab205718). The primary and secondary antibodies were diluted in the blocking solution (3% BSA, Sigma Chemical Co., St. Louis, MO, USA) at 1:3,000 v/v. The color associated with antigen labeling was developed using alkaline phosphatase (Bio-Rad Laboratories, Hercules, CA, 94547).^24^

## RESULTS

**Fig. 2:**
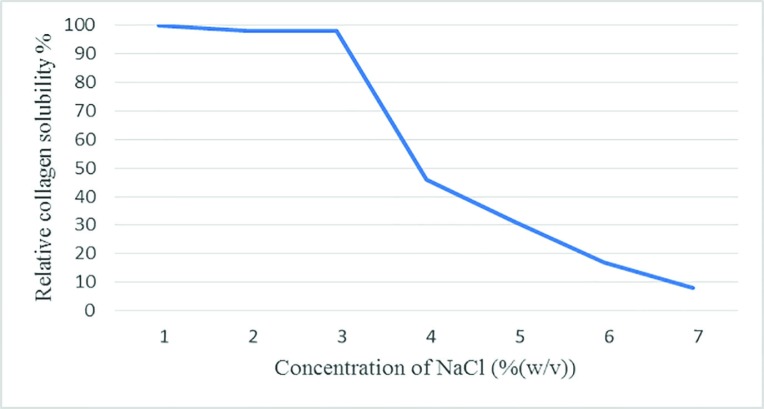
Relative solubility (%) of collagen in the presence of NaCl at different concentrations

**Fig. 3:**
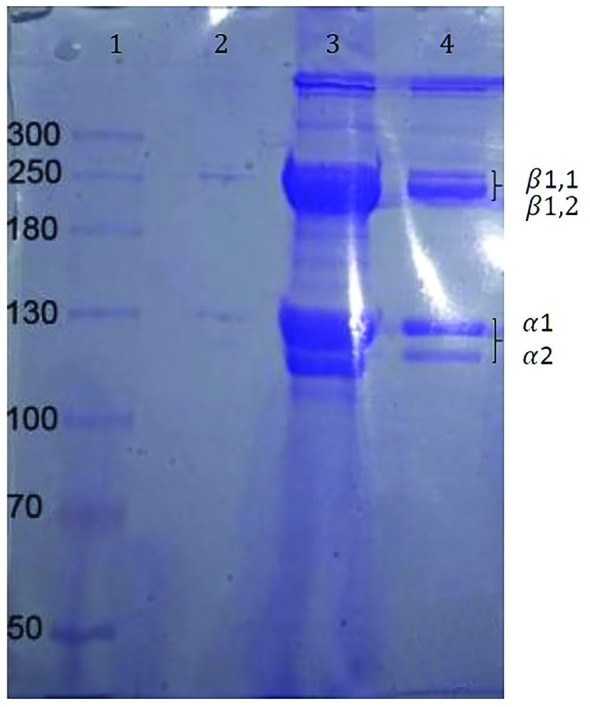
Electrophoretic migration patterns of control avian collagen and collagen type I solubilized using pepsin and ethylene diamine. Lane 1 contains Marker, lane 2 contains negative control, Lane 3 contains positive control using pepsin or ethylenediamine, and Lane 4 contains extracted collagen I solubilized using pepsin or ethylene diamine. β represents dimers, and α represents monomers of collagen chains

**Fig. 4:**
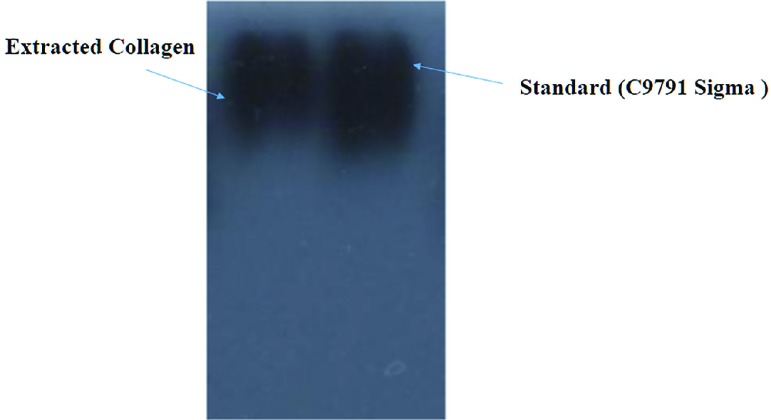
Western-blot identified using anti-His-Tag antibodies for collagen type I-expression

The solubility of collagen was sustained in the presence of NaCl up to 3%. Solubility decreased with a rise in NaCl concentration above 3%. The high concentration of NaCl caused a decrease in protein solubility by increasing hydrophobic interaction with the protein (Figure 2). SDS PAGE of extracted collagen type I was shown in Figure 3 and also, there was a control commercial collagen type I. The migration shapes of pepsin I and ethylene diamine I produced trimmers, dimers, and monomers (*α*b1 and *α*2). Evaluation of the electrophoresis showed the shape of collagen from extract pepsin I with ethylene diamine I, and also, the *α*- and *β*-chains of extracted collagen type I did not migrate quite as far in the acrylamide gel (Figure 3). Western blot analysis demonstrated that no obvious differences were visible in the amount of collagen type I between extracted and standard collagen and no significant difference was noted between extracted collagen form human placenta and standard collagen (Figure 4).

## DISCUSSION

Collagen is one of the commercial products derived from different parts of animals such as skin, bone, placenta and so on. Collagen contains 30 percent of organism proteins and has 19 variations designated as type I–XIX.^10^^,^^25^^,^^26^ Collagen causes an improvement in the strength and resistance of the tissue and also, is a main component of extracellular matrix (ECM).^27^^,^^28^ Collagen has been used widely in different part of industry such as food, medicine, cosmetics, cell culture, leather, film, pharmaceutical, and biomedical materials. Most commercial collagen products are extracted from pigs and cows. Transferring the diseases to humans can happen from animal extracted collagen sources such as BSE, and foot and mouth disease. Consumption request is increasing year to year and a new approach is needed for extracting of collagen with cost-effective, safe and a fast method.^29^

Studies have shown that bone collagen extraction was more tolerant of salt than skin collagen (NaCl at 4–6% concentration). Therefore, collagen extraction has various molecular properties which lead to different characteristics.^29^ Fish collagen has lower stability than mammalian collagen due to a lower imino acid in fish collagens.^28^^,^^30^ Collagen has been extracted from various animal parts and characterized.^11^^,^^25^^-^^28^^,^^30^. In this study, the human placenta was selected as the best part of human waste, which has abundant collagen and can be extracted from the placenta for clinical usage such as tissue engineering, biomedical purposes, stem therapy and research, etc. The new mixed, simple, fast and safe approach was obtained and by SDS page and western blot were confirmed. Martínez *et al.* performed research on extraction and characterization of rabbit skin collagen as a new alternative for collagen type I. They used acetic acid and pepsin for the extraction of soluble and insoluble collagens. They found that rabbit skin can be a good source for the collagen extraction.^25^

Cliché *et al.* studied on extraction and characterization of collagen from chicken skin. They extracted collagen with pepsin or ethylene diamine, while 38.9% of the collagen content in the solid phase was extracted with pepsin and 25.1% with ethylene diamine. They found that chicken skin can be a new source of high-quality collagen.^27^ Veeruraj *et al.* had research on extraction and characterization of acid soluble collagen and pepsin soluble collagen from the skin wastes of marine eel fish (Evenchelys Macrura) and they found a high solubility in acidic pH (1–4) and NaCl at concentration up to 3.0 and 4.0 percent (w/v) for ASC and PSC, respectively. 

Also, they suggested that the marine eel fish skin collagen could be used in the biomedical materials, food and pharmaceutical industries as a new source.^28^ Shah *et al.* studied on collagen extraction from the placenta of buffalo by acid solubilization with pepsin and they found that the placenta collagens were characterized as type I collagen containing α1 chains and one α2 chain with no disulfide bond and also, they concluded that the placenta of buffalo could be used as a potential source for biomaterial purposes.^26^

 Kittiphattanabawon *et al.* researched on extraction and characterization of ASC and PSC from the skin of brown-banded bamboo shark (Chiloscyllium Punctatum) and they found that collagen contained α and β chains as their major components and they concluded that the skin of brown-banded bamboo shark can be a new source of collagen for various applications.^30^ In this study, 0.06 M acetic acid (1:25(w/v)) and NaCl (3%, pH=7) were used for precipitating collagen. The solubility of collagens showed that the best concentration of NaCl was 3% and this concentration was used for final collagen extraction**. **The *α*- and *β*-chains of extracted collagen type I was identified and compered with control commercial collagen type I by SDS page. There was no significant difference between the extracted collagen and standard group. And also, this finding was confirmed by western blot test and it showed that there was no significant difference between standard and extracted collagen. 

It can be concluded that this new approach can be a novel method for extracting collagen from the placenta or other soft tissues. The criteria (such as cost-effectiveness, being safe and fast) causes that this method to be known as the selected method for extracting collagen from the human placenta for biomedical applications. 
